# Data related to conformation dependence of tyrosine binding on the surface of graphene: Bent prefers over parallel orientation

**DOI:** 10.1016/j.dib.2019.104420

**Published:** 2019-08-22

**Authors:** Dalia Daggag, Jovian Lazare, Tandabany Dinadayalane

**Affiliations:** Department of Chemistry, Clark Atlanta University, 223 James P. Brawley Drive, S.W, Atlanta, GA 30314, USA

**Keywords:** Graphene, Binding energy, HOMO, LUMO, HOMO-LUMO gap, Tyrosine, Mulliken charges

## Abstract

In this data article, M06-2X/6-31G(d) level optimized geometries of complexes of tyrosine conformers binding with graphene sheets are shown in top and side views with selected non-bonding distances. The images of frontier molecular orbitals from HOMO-15 to LUMO+15 of the complexes involving graphene with tyrosine conformers are presented and the isovalue is 0.003 au. For some complexes involving small graphene, the orbitals are from HOMO-5 to LUMO+5. The molecular orbitals highlighted with frames show obvious overlaps between the fragments. Total energies of small and large graphene (**G**_**S**_ and **G**_**L**_) and selected tyrosine conformers in gas and aqueous phases obtained at M06-2X/6-31G(d) level are given. The data also include total energies of all complexes in the gas phase and the aqueous phase, binding energies, BSSE (basis set superposition error) correction, and BSSE-corrected binding energies in gas phase and solvation effect on the binding energies obtained at M06-2X/6-31G(d) level. Mulliken charges of tyrosine conformers in gas and aqueous phases, and the deformation energy for tyrosine and graphene in the gas phase complexes are provided. The values of the highest occupied molecular orbital (HOMO), the lowest unoccupied molecular orbital (LUMO) and HOMO-LUMO energy gaps for some of graphene-tyrosine complexes that were not reported in the article [1] are given. The data is related to the research article “Conformation dependence of tyrosine binding on the surface of graphene: Bent prefers over parallel orientation” [1].

**Table:** Specifications Table

Subject area	*Materials Science*
More specific subject area	*Materials Chemistry*
Type of data	*Scheme, Figures and Tables*
How data was acquired	*Quantum Mechanical Calculations using Gaussian 09 software*
Data format	*Obtained results, analyzed, plotted the surfaces*
Experimental factors	*Computational experiments were done in gas and aqueous phases*
Experimental features	*DFT calculations for full geometry optimizations at M06-2X/6-31G(d) level; PCM for aqueous phase calculations;* HOMO and LUMO energies at TPSSh/6-31G(d)//M06-2X/6-31G(d) level
Data source location	*Department of Chemistry, Clark Atlanta University, Atlanta, Georgia, USA. Contact corresponding author if anyone needs data of output file.*
Data accessibility	*Data are available in this article*
Related research article	D. Daggag, J. Lazare, T. Dinadayalane, Conformation Dependence of Tyrosine Binding on the Surface of Graphene: Bent Prefers Over Parallel Orientation. Applied Surface Science, 483 (2019) 178–186 [1].

**Table:** 

**Value of the Data** • This data will be useful for the scientists working in the area of graphene-based materials for biosensors. • The top and side views of the complexes with the non-bonding interactions will be helpful to understand the strength of complexes of different orientations of tyrosine with graphene. • Total energies, HOMO, LUMO and HOMO-LUMO energy gap values will be useful for others to compare their computational results and the later values could be compared to the future experimental data. • The pictures of frontier molecular orbitals of the complexes will be helpful for physical organic chemists to understand the intermolecular orbital interactions when the orientations and the conformers of tyrosine change on the surface of graphene.

## 1 Data

**Scheme 1:**
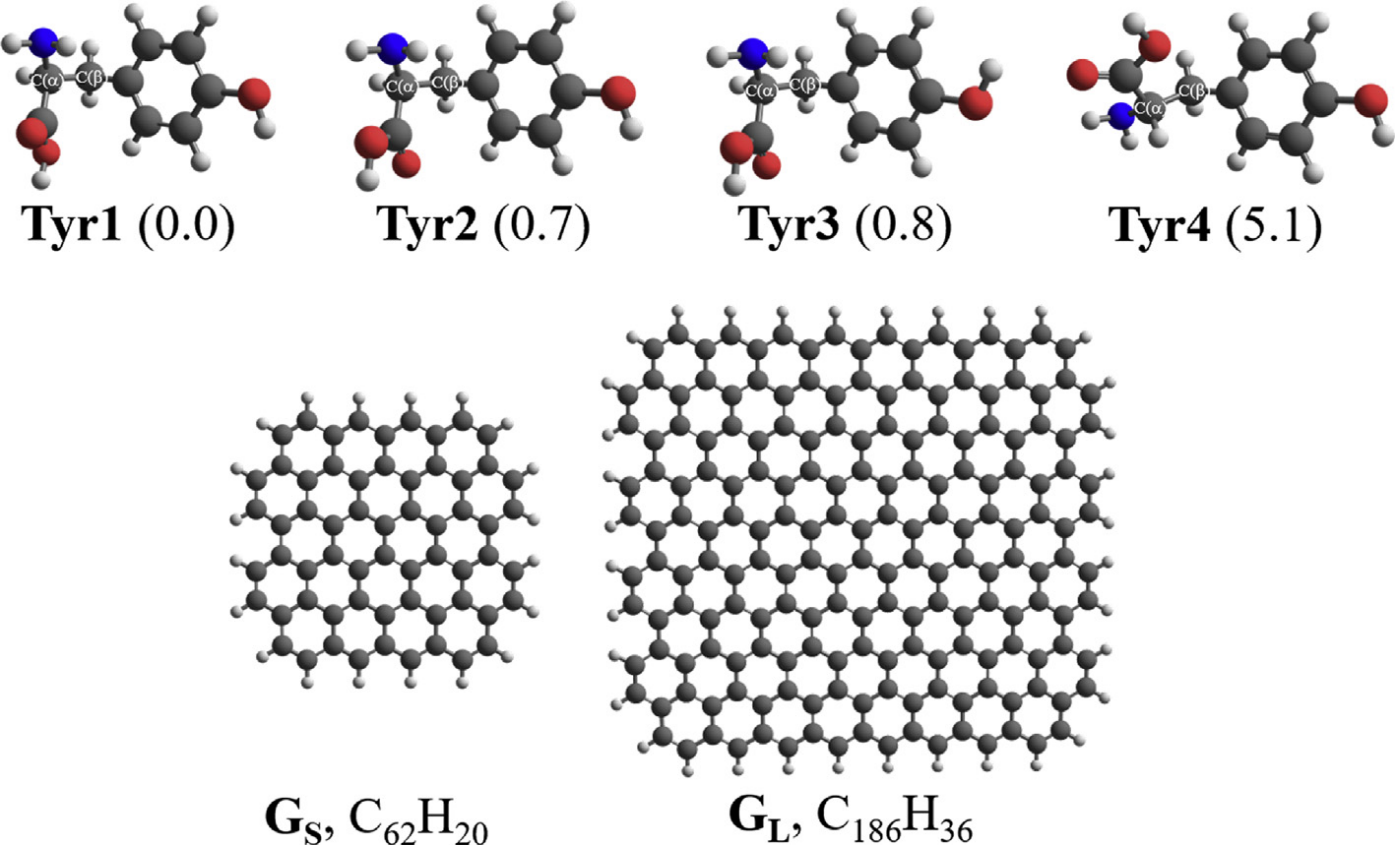
M06-2X/6-31G(d) level optimized structures of three most stable conformers (**Tyr1**, **Tyr2**, and **Tyr3**) and one of the high energy conformers (**Tyr4)** of tyrosine along with the relative energy (in kcal/mol), and two finite size graphene sheets of small graphene (**G**_**S**_) consisting of 62 carbon atoms and large graphene sheet (**G**_**L**_) consisting of 186 carbon atoms.

**Fig. 1:**
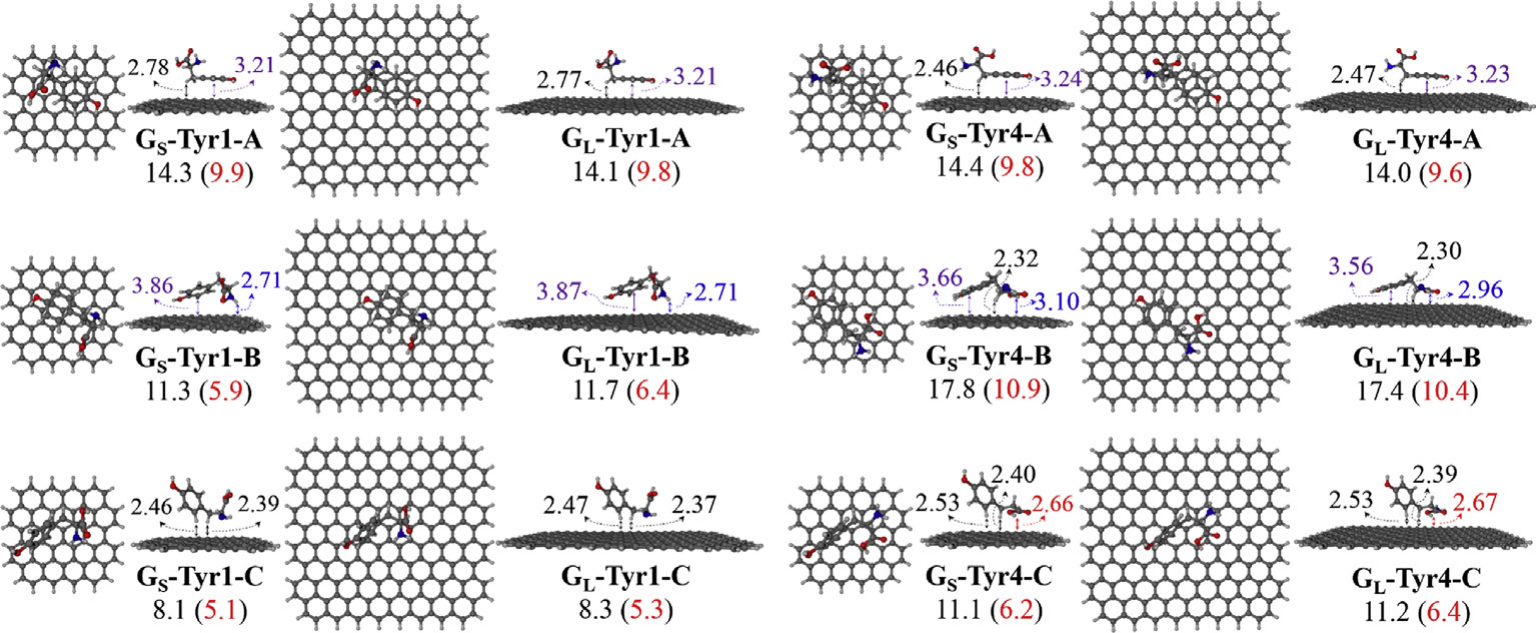
M06-2X/6-31G(d) level optimized geometries of complexes of tyrosine (**Tyr1** and **Tyr4**) binding with graphene sheets (**G**_**S**_ and **G**_**L**_). Showing top and side views with selected non-bonding distances in Å for C–H^…^π (in black), π−π (in purple), O–H^…^π (in red), and/or N–H^…^π (in blue) interactions, and the binding energies in the gas phase. The values given in parentheses correspond to the BSSE corrected binding energies. All values of binding energies are in kcal/mol.

**Fig. 2:**
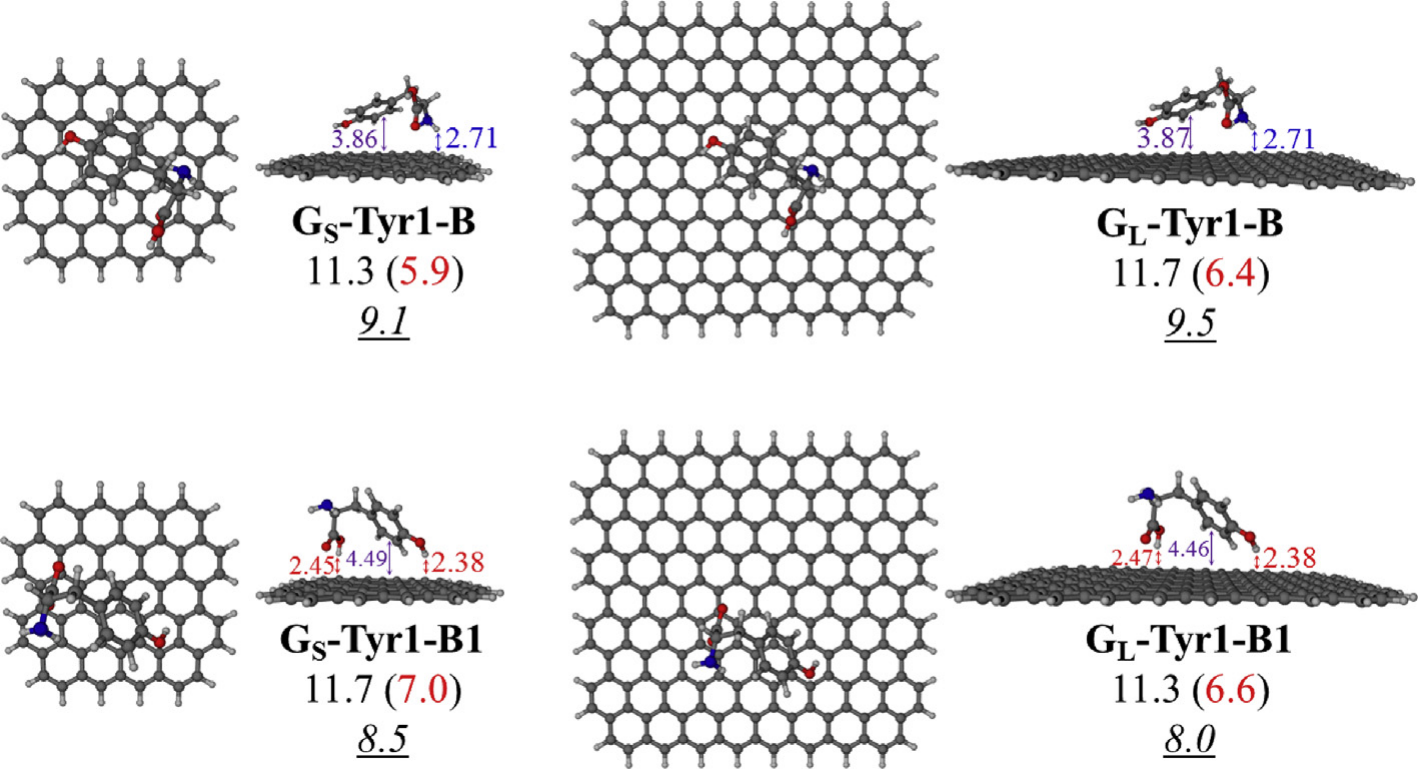
M06-2X/6-31G(d) level optimized geometries of complexes **G**_**S/L**_**-Tyr1-B** and **G**_**S/L**_**-Tyr1-B1** showing selected non-bonding distances (in Å). The values of binding energies in gas phase without and with (in parentheses) BSSE correction are provided. The values of binding energies in aqueous phase are given in underlined and italics. All values of binding energies are in kcal/mol. The complexes **G**_**S/L**_**-Tyr1-B** exhibit strong N–H^…^π (in blue) interactions whereas **G**_**S/L**_**-Tyr1-B1** show predominant O–H^…^π (in red) interactions.

**Fig. 3:**
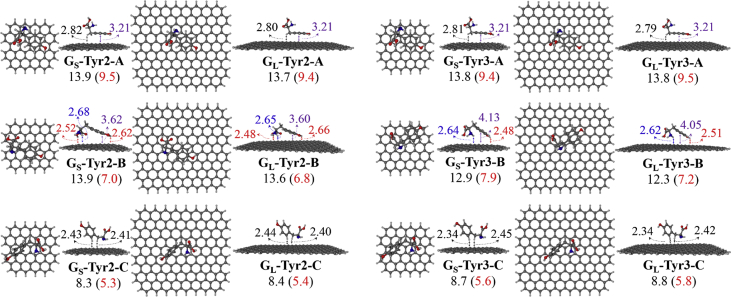
M06-2X/6-31G(d) level optimized geometries of complexes of tyrosine (**Tyr2** and **Tyr3**) binding with graphene sheets (**G**_**S**_ and **G**_**L**_). Showing top and side views with selected non-bonding distances in Å for C–H^…^π (in black), π−π (in purple), O–H^…^π (in red), and/or N–H^…^π (in blue) interactions, and the binding energies in the gas phase. The values given in parentheses correspond to the BSSE corrected binding energies. All values of binding energies are in kcal/mol.

**Fig. 4:**
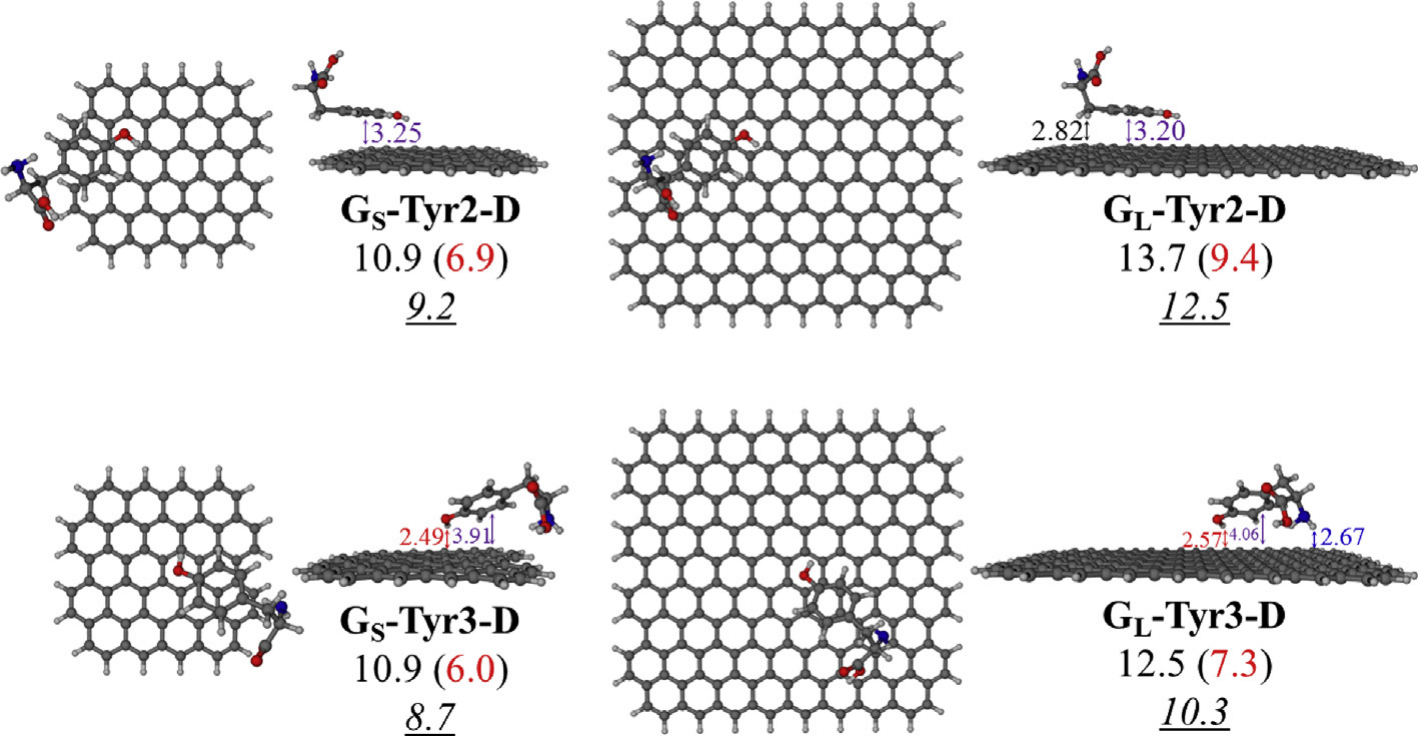
M06-2X/6-31G(d) level optimized geometries of the complexes **G**_**S/L**_**-Tyr2-D** and **G**_**S/L**_**-Tyr3-D** showing the amino acids near the edges of graphene. Selected non-bonding distances (in Å) and the binding energies in gas phase without and with (in parentheses) BSSE correction are provided. The values of binding energies in aqueous phase are given in underlined and italics. All values of binding energies are in kcal/mol.

**Fig. 5:**
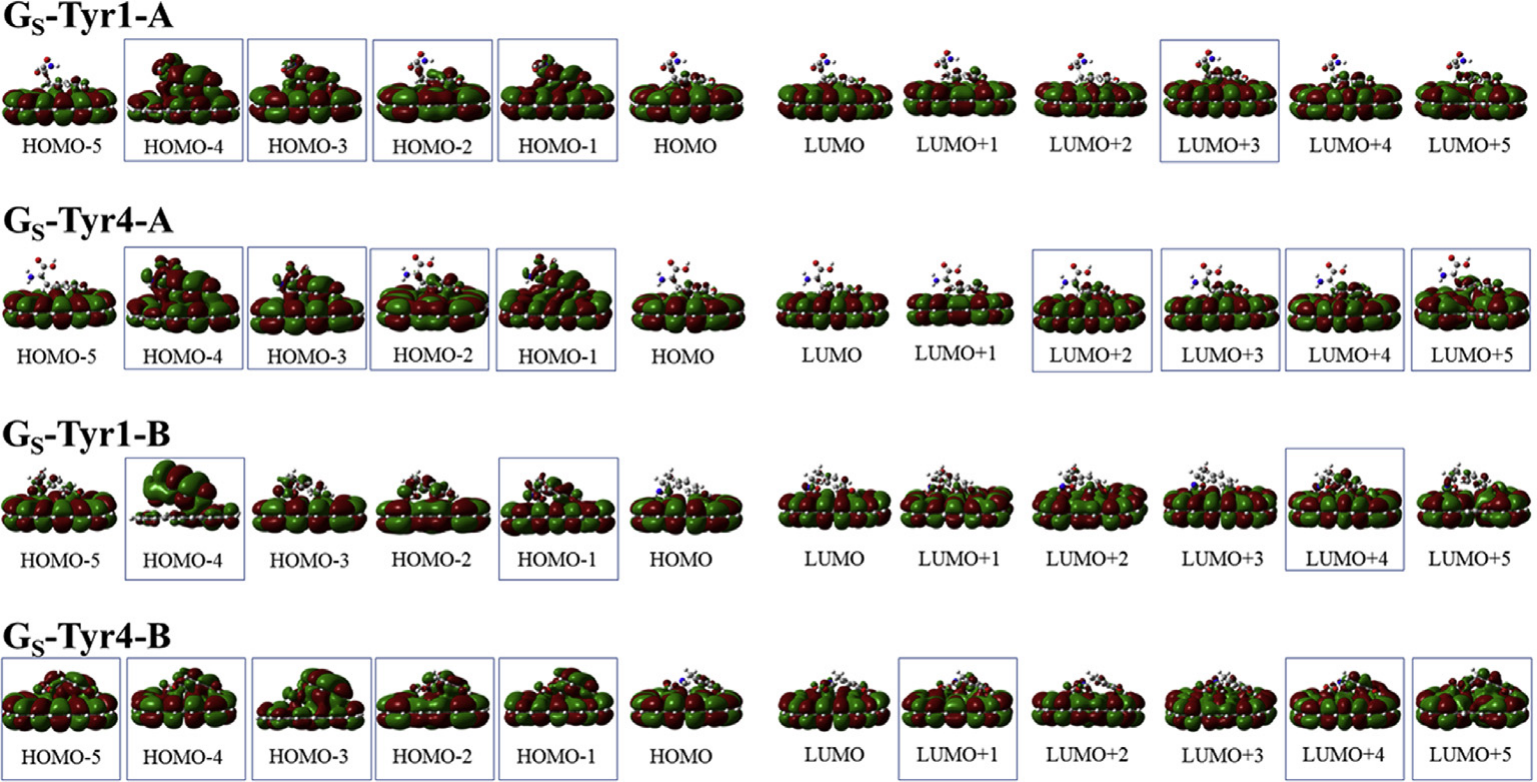
The pictorial presentations of frontier molecular orbitals from HOMO-5 to LUMO+5 (isovalue = 0.003 au) of the complexes **G**_**S**_**-Tyr1-A**, **G**_**S**_**-Tyr4-A**, **G**_**S**_**-Tyr1-B**, and **G**_**S**_**-Tyr4-B**. The molecular orbitals highlighted with frames show obvious overlaps between the fragments.

**Fig. 6:**
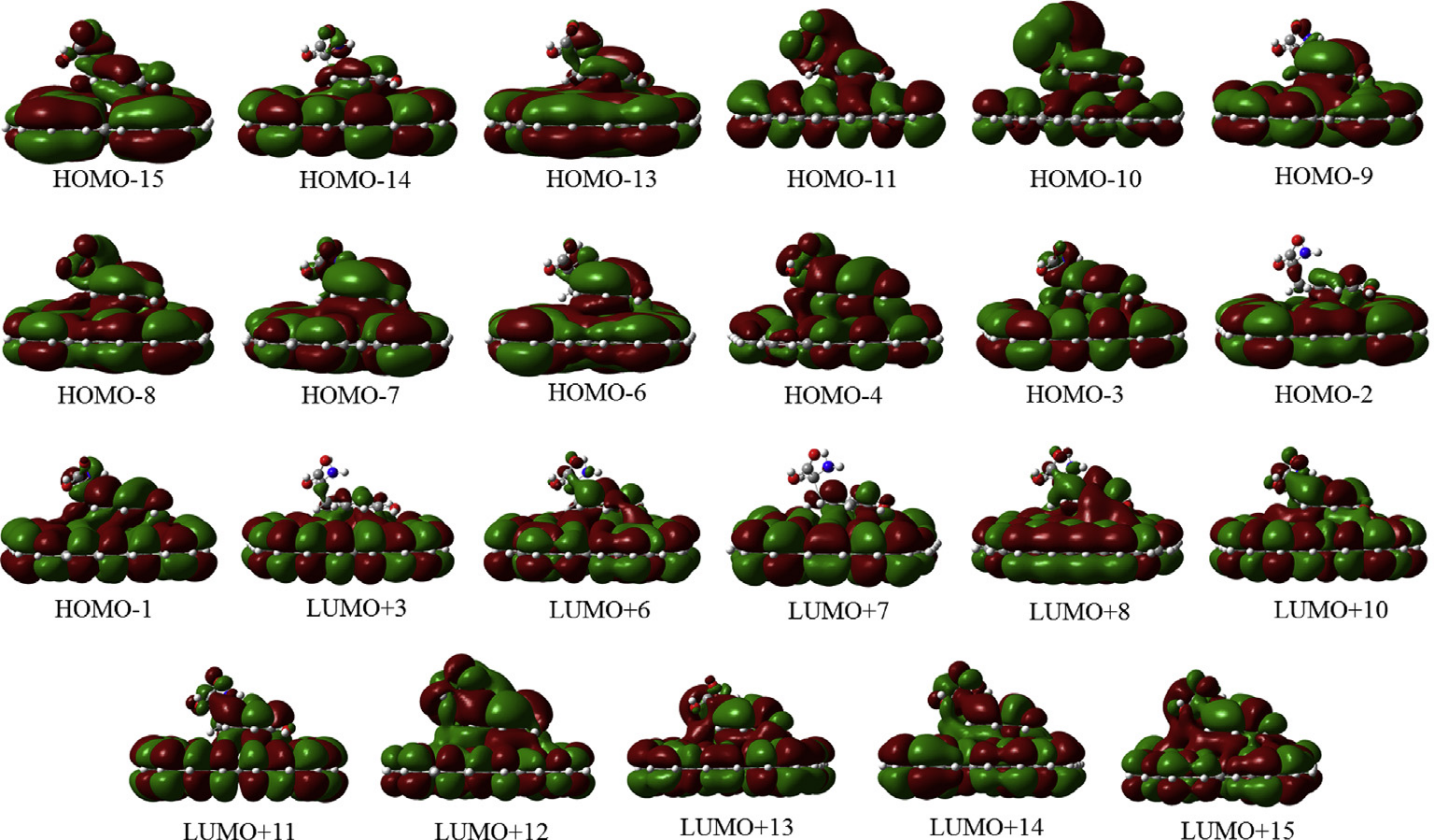
The pictures of selected frontier molecular orbitals for the complex **G**_**S**_**-Tyr1-A** showing obvious overlaps between the fragments. The orbitals are from HOMO-15 to LUMO+15 and the isovalue is 0.003 au.

**Fig. 7:**
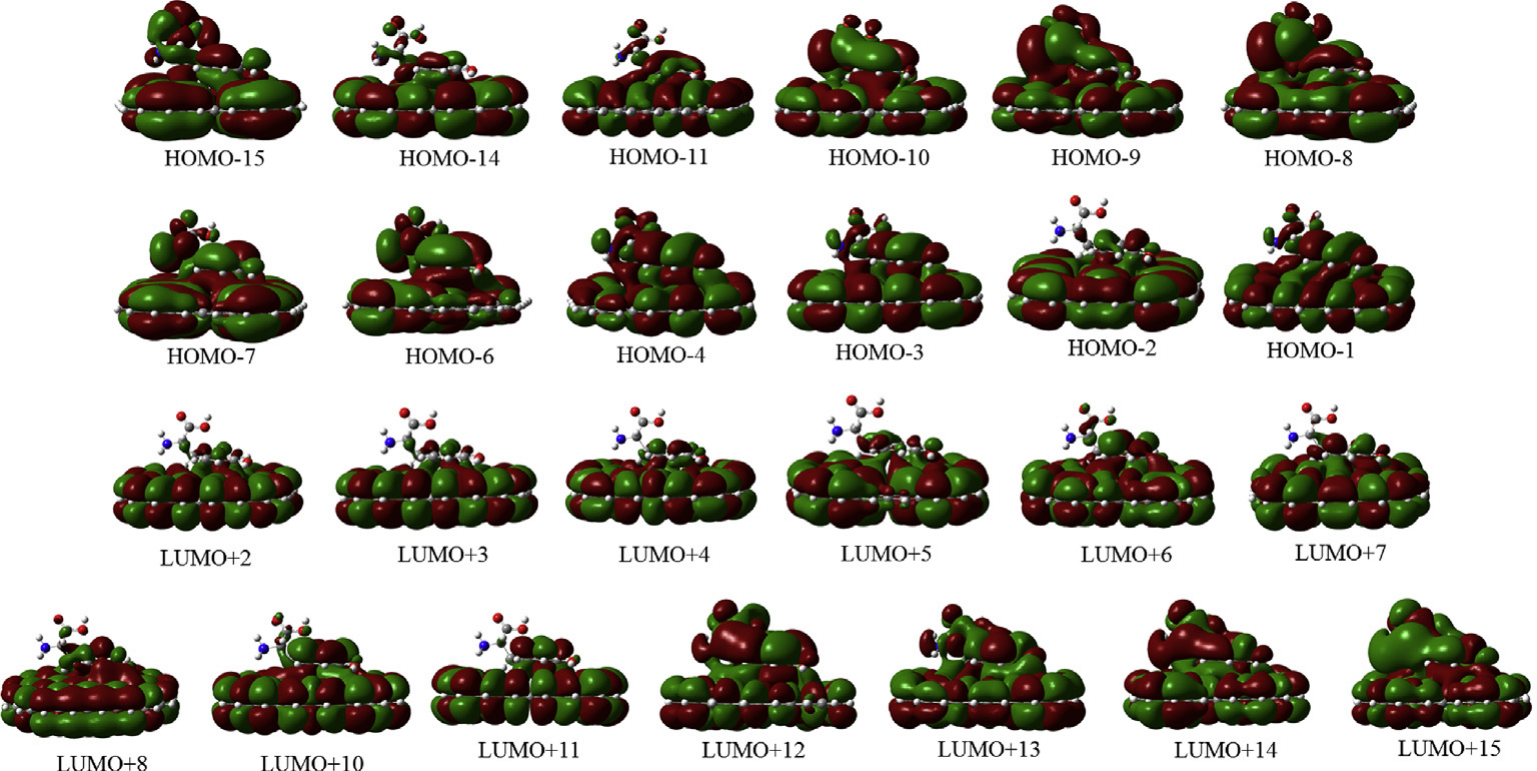
The pictures of selected frontier molecular orbitals for the complex **G**_**S**_**-Tyr4-A** showing obvious overlaps between the fragments. The orbitals are from HOMO-15 to LUMO+15 and the isovalue is 0.003 au.

**Fig. 8:**
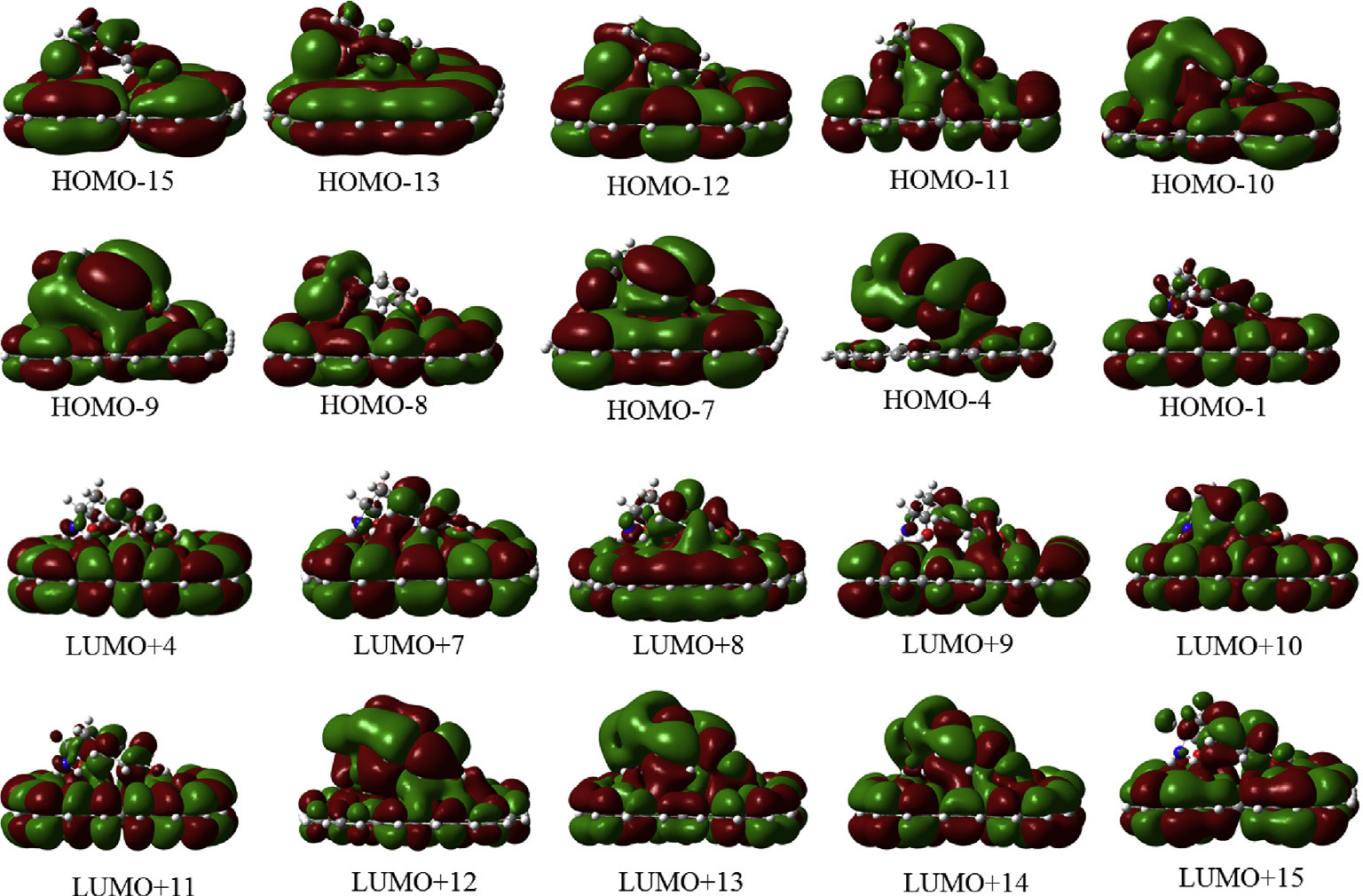
The pictures of selected frontier molecular orbitals for the complex **G**_**S**_**-Tyr1-B** showing obvious overlaps between the fragments. The orbitals are from HOMO-15 to LUMO+15 and the isovalue is 0.003 au.

**Fig. 9:**
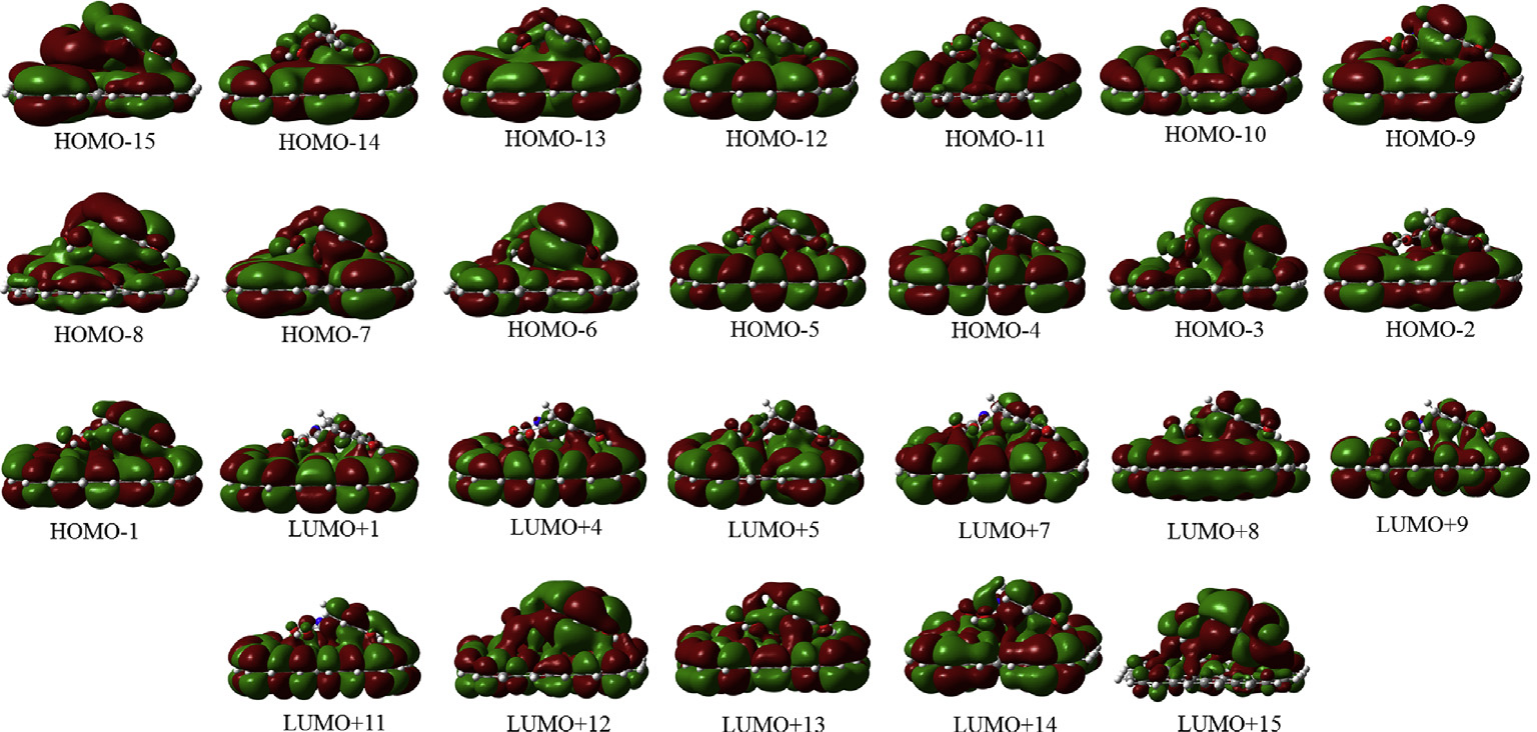
The pictures of selected frontier molecular orbitals for the complex **G**_**S**_**-Tyr4-B** showing obvious overlaps between the fragments. The orbitals are from HOMO-15 to LUMO+15 and the isovalue is 0.003 au.

**Fig. 10:**
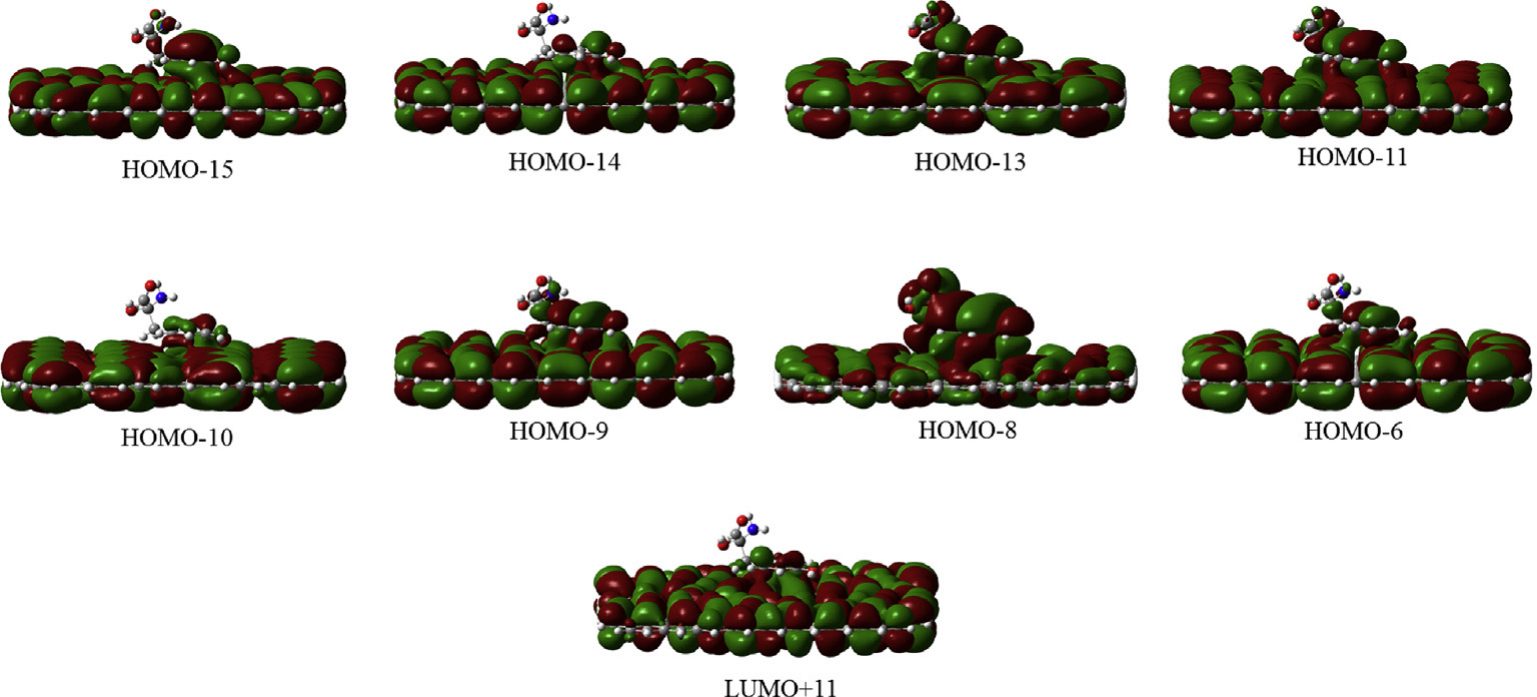
The pictures of selected frontier molecular orbitals for the complex **G**_**L**_**-Tyr1-A** showing obvious overlaps between the fragments. The orbitals are from HOMO-15 to LUMO+15 and the isovalue is 0.003 au.

**Fig. 11:**
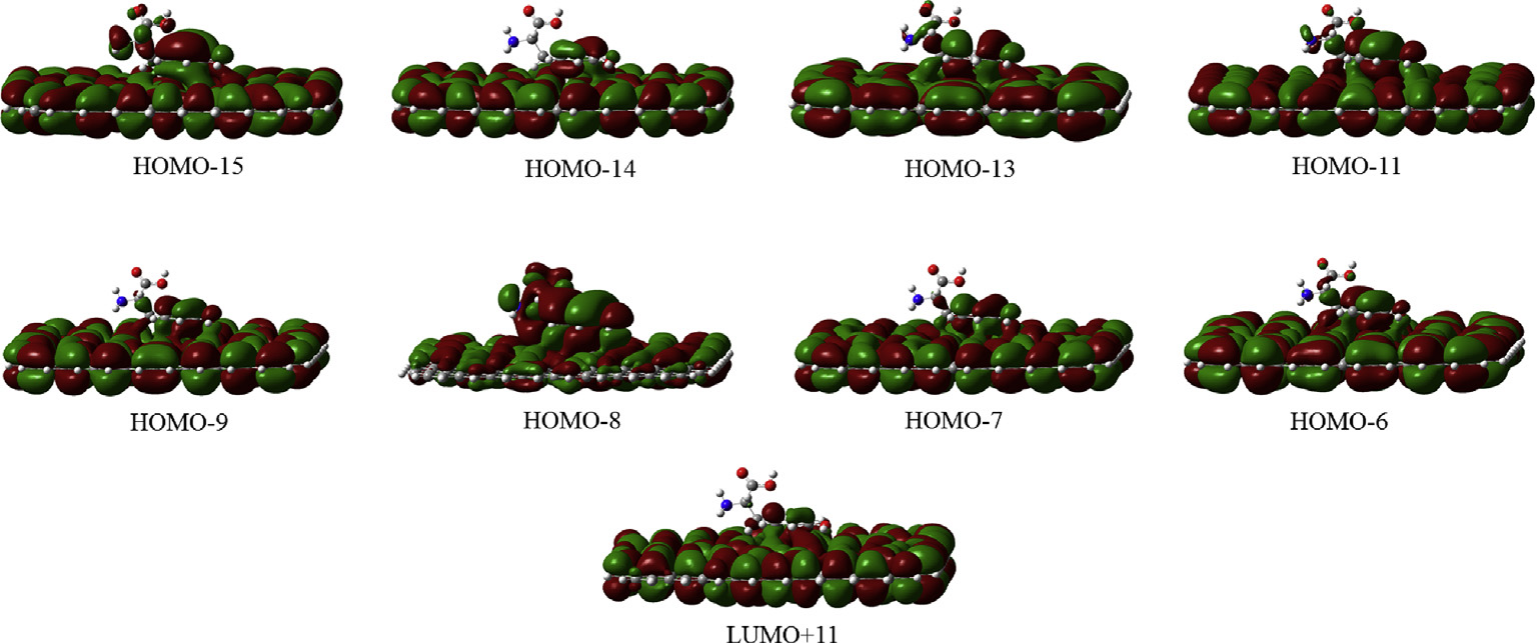
The pictures of selected frontier molecular orbitals for the complex **G**_**L**_**-Tyr4-A** showing obvious overlaps between the fragments. The orbitals are from HOMO-15 to LUMO+15 and the isovalue is 0.003 au.

**Fig. 12:**
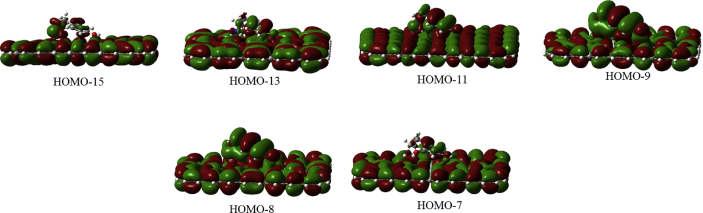
The pictures of selected frontier molecular orbitals for the complex **G**_**L**_**-Tyr1-B** showing obvious overlaps between the fragments. The orbitals are from HOMO-15 to LUMO+15 and the isovalue is 0.003 au. No orbital from LUMO shows the obvious overlap.

**Fig. 13:**
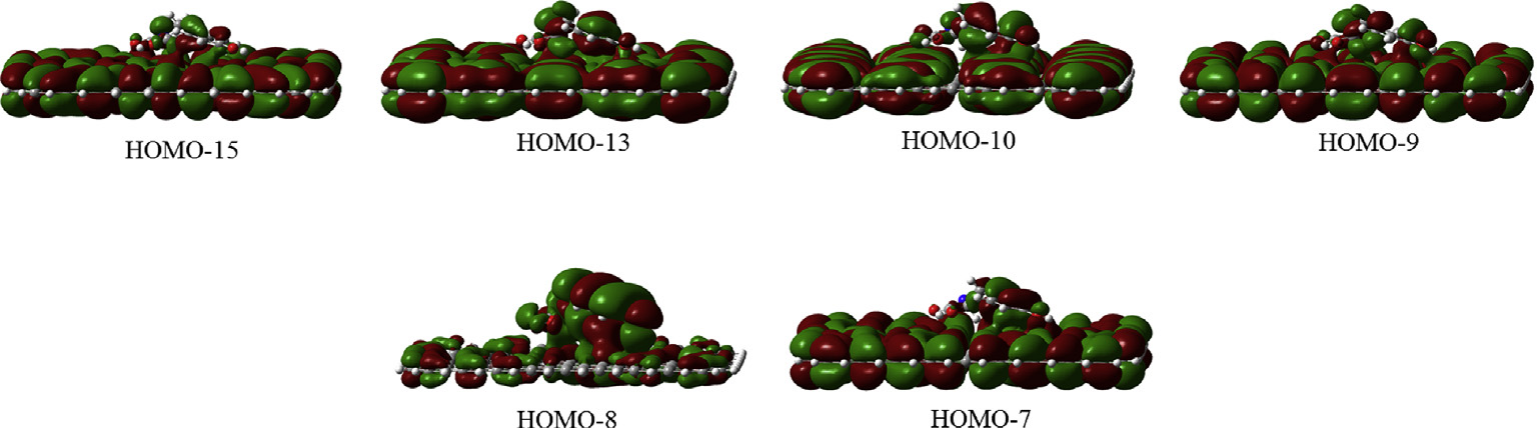
The pictures of selected frontier molecular orbitals for the complex **G**_**L**_**-Tyr4-B** showing obvious overlaps between the fragments. The orbitals are from HOMO-15 to LUMO+15 and the isovalue is 0.003 au. No orbital from LUMO shows the obvious overlap.

Four selected conformers of tyrosine (Tyr) with two different sizes of the graphene sheets are shown in Scheme 1. Fig. 1 shows top and side views with selected non-bonding distances and the binding energies for the complexes of tyrosine (**Tyr1** and **Tyr4** conformers) binding with graphene sheets (**G**_**S**_ and **G**_**L**_) at M06-2X/6-31G(d) level. Fig. 2 depicts M06-2X/6-31G(d) level optimized geometries of complexes having different bent orientations of **Tyr1** conformer in **G**_**S/L**_**-Tyr1-B** and **G**_**S/L**_**-Tyr1-B1** with selected non-bonding distances and binding energies. Fig. 3 displays top and side views of the complexes having **Tyr2** and **Tyr3** conformers on graphene surfaces (**G**_**S**_ and **G**_**L**_) obtained at M06-2X/6-31G(d) level and the selected non-bonding distances as well as the binding energies are provided. The complexes **G**_**S/L**_**-Tyr2-D** and **G**_**S/L**_**-Tyr3-D** showing the amino acids near the edges of graphene are depicted in Fig. 4 wherein the selected non-bonding distances and binding energies are provided. Fig. 5 shows the images of frontier molecular orbitals (FMOs) from HOMO-5 to LUMO+5 of the complexes **G**_**S**_**-Tyr1-A**, **G**_**S**_**-Tyr4-A**, **G**_**S**_**-Tyr1-B**, and **G**_**S**_**-Tyr4-B**. Fig. 6 and Fig. 7 display the pictures of selected FMOs from HOMO-15 to LUMO+15 showing obvious overlaps between the fragments for the complexes **G**_**S**_**-Tyr1-A** and **G**_**S**_**-Tyr4-A**, respectively. Fig. 8 and Fig. 9 display the pictures of selected FMOs from HOMO-15 to LUMO+15 showing obvious overlaps between the fragments for the complexes **G**_**S**_**-Tyr1-B** and **G**_**S**_**-Tyr4-B**, respectively. The pictures of selected FMOs from HOMO-15 to LUMO+15 showing obvious overlaps between the fragments for the complexes **G**_**L**_**-Tyr1-A** and **G**_**L**_**-Tyr4-A**, **G**_**L**_**-Tyr1-B** and **G**_**L**_**-Tyr4-B** are presented in Fig. 10, Fig. 11, Fig. 12 and Fig. 13, respectively. Table 1 lists total energies of graphene and selected tyrosine conformers in gas and aqueous phases. Table 2 provides total energies of all complexes in the gas and the aqueous phases, binding energies without and with BSSE correction, and solvation effect on binding energies obtained at M06-2X/6-31G(d) level. Table 3 provides Mulliken charges of **Tyr** in gas and aqueous phases, and the deformation energy for **Tyr** and graphene in the gas phase for selected complexes. Table 4 lists the values of HOMO and LUMO energies, and HOMO-LUMO energy gaps of selected graphene-tyrosine complexes in the gas phase at the TPSSh/6-31G(d)//M06-2X/6-31G(d) level.

**Table 1:** Total energies (in hartrees) of graphene (**G**_**S**_ and **G**_**L**_) and selected tyrosine conformers (**Tyr1**, **T**y**r2**, **Tyr3** and **Tyr4**) in gas (*E*) and aqueous phases (*E*_aq_) obtained at M06-2X/6-31G(d) level.

Structure	Total *E* (hartrees)	Total *E*_aq_ (hartrees)
G_S_	−2374.17243	−2374.18661
G_L_	−7108.47660	−7108.50375
Tyr1	−629.75085	−629.76529
Tyr2	−629.74970	−629.76350
Tyr3	−629.74965	−629.76358
Tyr4	−629.74267	−629.75845

**Table 2:** Total energies (in hartrees) of all complexes in the gas phase (*E*) and the aqueous phase (*E*_aq_), binding energies (Δ*E* and Δ*E*_aq_), BSSE correction, and BSSE-corrected binding energies in gas phase (Δ*E*_BSSE_) and solvation effect on binding energies obtained at M06-2X/6-31G(d) level. Except total energies, all other values are in kcal/mol.

Complex (X = S or L)	Total *E* (hartrees)	Δ*E* (kcal/mol)	BSSE correction (kcal/mol)	Δ*E*_BSSE_ (kcal/mol)	Total *E*_aq_ (hartrees)	Δ*E*_aq_ (kcal/mol)	Solvation effect (kcal/mol)
G_S_-Tyr1-A	−3003.94600	14.3	4.4	9.9	−3003.97155	12.3	−2.0
G_S_-Tyr1-B	−3003.94130	11.3	5.4	5.9	−3003.96648	9.1	−2.2
G_S_-Tyr1-B1	−3003.94187	11.7	4.7	7.0	−3003.96548	8.5	−3.2
G_S_-Tyr1-C	−3003.93624	8.1	3.0	5.1	−3003.96252	6.7	−1.4
G_S_-Tyr2-A	−3003.94426	13.9	4.4	9.5	−3003.96986	12.4	−1.5
G_S_-Tyr2-B	−3003.94435	13.9	6.9	7.0	−3003.96769	11.0	−2.9
G_S_-Tyr2-C	−3003.93538	8.3	3.0	5.3	−3003.96096	6.8	−1.5
G_S_-Tyr2-D	−3003.93951	10.9	4.0	6.9	−3003.96481	9.2	−1.7
G_S_-Tyr3-A	−3003.94415	13.8	4.4	9.4	−3003.96997	12.4	−1.4
G_S_-Tyr3-B	−3003.94261	12.9	5.0	7.9	−3003.96687	10.5	−2.4
G_S_-Tyr3-C	−3003.93600	8.7	3.1	5.6	−3003.96098	6.8	−1.9
G_S_-Tyr3-D	−3003.93951	10.9	4.9	6.0	−3003.96400	8.7	−2.2
G_S_-Tyr4-A	−3003.93807	14.4	4.6	9.8	−3003.96477	12.4	−2.0
G_S_-Tyr4-B	−3003.94344	17.8	6.9	10.9	−3003.96936	15.2	−2.6
G_S_-Tyr4-C	−3003.93275	11.1	4.9	6.2	−3003.95919	8.9	−2.2
G_L_-Tyr1-A	−7738.24986	14.1	4.3	9.8	−7738.28911	12.6	−1.5
G_L_-Tyr1-B	−7738.24603	11.7	5.3	6.4	−7738.28417	9.5	−2.2
G_L_-Tyr1-B1	−7738.24546	11.3	4.7	6.6	−7738.28177	8.0	−3.3
G_L_-Tyr1-C	−7738.24061	8.3	3.0	5.3	−7738.28000	6.9	−1.4
G_L_-Tyr2-A	−7738.24821	13.7	4.3	9.4	−7738.28729	12.6	−1.1
G_L_-Tyr2-B	−7738.24794	13.6	6.8	6.8	−7738.28930	13.8	0.2
G_L_-Tyr2-C	−7738.23968	8.4	3.0	5.4	−7738.27838	7.0	−1.4
G_L_-Tyr2-D	−7738.24810	13.7	4.3	9.4	−7738.28721	12.5	−1.2
G_L_-Tyr3-A	−7738.24818	13.8	4.3	9.5	−7738.28739	12.6	−1.2
G_L_-Tyr3-B	−7738.24587	12.3	5.1	7.2	−7738.28348	10.1	−2.2
G_L_-Tyr3-C	−7738.24027	8.8	3.0	5.8	−7738.27844	7.0	−1.8
G_L_-Tyr3-D	−7738.24623	12.5	5.2	7.3	−7738.28374	10.3	−2.2
G_L_-Tyr4-A	−7738.24158	14.0	4.4	9.6	−7738.28197	12.4	−1.6
G_L_-Tyr4-B	−7738.24704	17.4	7.0	10.4	−7738.28560	14.7	−2.7
G_L_-Tyr4-C	−7738.23719	11.2	4.8	6.4	−7738.27665	9.1	−2.1

**Table 3:** Mulliken charges (in e^−^) of **Tyr** in gas phase (aqueous phase), and the deformation energy (Δ*E*_def_, kcal/mol) for **Tyr** and graphene in the gas phase of selected complexes.

Complex (X = S or L)	X = S (Small system)			X = L (Large system)		
	Mulliken charges of Tyr (e^−^)	Δ*E*_def_ of Tyr (kcal/mol)	Δ*E*_def_ of G_S_ (kcal/mol)	Mulliken charges of Tyr (e^−^)	Δ*E*_def_ of Tyr (kcal/mol)	Δ*E*_def_ of G_L_ (kcal/mol)
G_X_-Tyr1-B1	−0.034 (−0.038)	0.23	0.09	−0.029 (−0.031)	0.24	0.07
G_X_-Tyr2-D	−0.009 (−0.009)	0.28	0.13	−0.008 (−0.009)	0.16	0.17
G_X_-Tyr3-D	−0.019 (−0.020)	0.40	0.28	−0.018 (−0.020)	0.45	0.06

**Table 4:** The values of HOMO (*E*_HOMO_), LUMO (*E*_LUMO_) and HOMO-LUMO energy gaps (*E*_g_) of selected graphene-tyrosine complexes in the gas phase at the TPSSh/6-31G(d)//M06-2X/6-31G(d) level. All values are in eV.

Complex (X = S or L)	X = S (Small system)			X = L (Large system)		
	*E*_HOMO_ (eV)	ELUMO (eV)	E_g_ (eV)	EHOMO (eV)	ELUMO (eV)	E_g_ (eV)
G_x_-Tyr1-B1	−4.34	−2.90	1.44	−3.76	−3.67	0.09
G_X_-Tyr2-D	−4.26	−2.83	1.43	−3.72	−3.64	0.08
G_X_-Tyr3-D	−4.34	−2.91	1.43	−3.77	−3.69	0.08

## 2 Experimental design, materials, and methods

Selected conformers of tyrosine, two different sizes of graphene systems, and tyrosine-graphene complexes were fully optimized using M06-2X functional [2] with 6-31G(d) basis set. The corrections for basis set superposition error (BSSE) were calculated for gas phase complexes by using the counterpoise technique proposed by Boys and Bernardi [3]. M06-2X/6-31G(d) level was used to obtain the molecular orbital pictures of selected individual fragments and complexes to examine intermolecular orbital interactions. We performed single point energy calculations at the M06-2X/6-31G(d) level in the aqueous medium using the polarizable continuum model (PCM) [4], [5]. Single point calculations were performed using the TPSSh functional [6], [7], [8] with 6-31G(d) basis set to calculate the energies of the highest occupied molecular orbital (HOMO) and the lowest unoccupied molecular orbital (LUMO), and HOMO-LUMO energy gaps. All calculations were performed using Gaussian 09 program package [9].
